# Temporal Dynamics of Subclinical Malaria in Different Transmission Zones of Myanmar

**DOI:** 10.4269/ajtmh.22-0027

**Published:** 2022-07-25

**Authors:** Joseph R. Egger, Kay T. Han, Huang Fang, Xiao Nong Zhou, Tin M. Hlaing, Myo Thant, Zay Y. Han, Xiao X. Wang, Tu Hong, Alyssa Platt, Ryan Simmons, Thynn K. Thane, Manfred Meng, Joyce Hogue, Christine F. Markwalter, Aung Thi, Thura Htay, Zaw W. Thein, Aye K. Paing, Zin M. Tun, Swai M. Oo, Poe P. Aung, Myaing M. Nyunt, Christopher V. Plowe

**Affiliations:** ^1^ Duke Global Health Institute, Duke University, Durham, North Carolina;; ^2^ Department of Medical Research, Ministry of Health and Sports, Yangon, Myanmar;; ^3^ National Institute of Parasitic Diseases, Chinese Center for Disease Control and Prevention, Shanghai, China;; ^4^ Zhejiang Provincial Center for Disease Control and Prevention, Hangzhou, Zhejiang, China;; ^5^ Defense Services Medical Research Center, Nay Pyi Taw, Myanmar;; ^6^ PPD, Morrisville, North Carolina;; ^7^ National Malaria Control Program, Myanmar Ministry of Health and Sports, Nay Pyi Taw, Myanmar;; ^8^ Department of Medicine, University of Maryland School of Medicine, Baltimore, Maryland

## Abstract

Countries in the Greater Mekong Subregion have committed to eliminate *Plasmodium falciparum* malaria by 2025. Subclinical malaria infections that can be detected by highly sensitive polymerase chain reaction (PCR) testing in asymptomatic individuals represent a potential impediment to this goal, although the extent to which these low-density infections contribute to transmission is unclear. To understand the temporal dynamics of subclinical malaria in this setting, a cohort of 2,705 participants from three epidemiologically distinct regions of Myanmar was screened for subclinical *P. falciparum* and *P. vivax* infection using ultrasensitive PCR (usPCR). Standard rapid diagnostic tests (RDTs) for *P. falciparum* were also performed. Individuals who tested positive for malaria by usPCR were followed for up to 12 weeks. Regression analysis was performed to estimate whether the baseline prevalence of infection and the count of repeated positive tests were associated with demographic, behavioral, and clinical factors. At enrollment, the prevalence of subclinical malaria infection measured by usPCR was 7.7% (1.5% *P. falciparum* monoinfection, 0.3% mixed *P. falciparum* and *P. vivax*, and 6.0% *P. vivax* monoinfection), while *P. falciparum* prevalence measured by RDT was just 0.2%. Prevalence varied by geography and was higher among older people and in those with outdoor exposure and travel. No difference was observed in either the prevalence or count of subclinical infection by time of year, indicating that even in low-endemicity areas, a reservoir of subclinical infection persists year-round. If low-density infections are shown to represent a significant source of transmission, identification of high-risk groups and locations may aid elimination efforts.

## INTRODUCTION

In the last 20 years, the number of reported cases of *Plasmodium falciparum* malaria has decreased by 97% in the Greater Mekong Subregion, which is composed of Cambodia, China’s Yunnan Province, Laos, Myanmar, Thailand, and Vietnam.^1^ Myanmar, which until recently had the heaviest malaria burden in the region,^2^ has seen a more than 12-fold decrease in cases in the last decade, from nearly 700,000 reported cases in 2010 to just over 56,000 in 2019.^1^ On the basis of this progress, and to deter the emergence and spread of artemisinin-resistant *P. falciparum*, the WHO has recommended that falciparum malaria be eliminated in the Greater Mekong Subregion by 2025, and all malaria by 2030.^3^

One potential impediment to malaria elimination in the region is the large reservoir of low-density, generally asymptomatic malaria. Historically, most studies of asymptomatic malaria have relied on light microscopy or rapid diagnostic tests (RDTs), which have lower limits of detection (LoD) of about 50–100 parasites/mL (or 50,000–100,000/mL). More recent surveys in the Greater Mekong Subregion have used ultrasensitive polymerase chain reaction (usPCR) assays that have LoDs of 16–22 parasites/mL.^4^^,^^5^ Surveys using these newer molecular assays, with their hundreds—or even thousands-fold lower detection limits—have revealed an unexpectedly high prevalence of subclinical infection in low transmission settings targeted for elimination.^6^^–^^9^

The potential for these subclinical infections—especially low-density infections that can only be detected using usPCR—to contribute to transmission is uncertain, and the question of whether they should be targeted in elimination efforts remains unsettled. Scores of countries have eliminated malaria without specifically targeting low-density infections that are clinically silent, and transmission potential is strongly correlated with higher parasite densities, well above the LoDs for microscopy and RDTs. Still, some have called for treating all subclinical malaria infections,^10^^,^^11^ arguing that these infections increase morbidity to the infected host, and that today’s low-density infection can become tomorrow’s or next month’s higher-density infection associated with clinical illness and/or increased risk of transmission.

The initial studies describing high prevalence of subclinical malaria in the Greater Mekong Subregion were cross-sectional surveys,^6^^–^^9^ leaving open the question of whether these were transient infections that might resolve before reaching densities associated with illness and transmission, or if they can persist and increase in density over time. A recent longitudinal cohort study from Vietnam used usPCR to prospectively follow participants with subclinical infections. With monthly follow-up visits, the median duration of subclinical infection for *P. falciparum* and *P. vivax* infections was estimated to be 2 and 6 months, respectively,^12^ and participants with *P. falciparum* infection had a 20% risk of having parasitemia for 4 months or longer, which increased to 59% for *P. vivax* infections.

Previous studies from the region have identified risk factors for subclinical malaria infection, including age (young adults) and male gender,^6^^,^^13^ as well as occupations that involve outdoor exposure, such as farming and logging.^13^^,^^14^ In preparation for a larger cohort study aiming to measure the transmission potential of subclinical malaria, we evaluated the dynamics of subclinical malaria over a period of 6–12 weeks, using weekly or biweekly follow-up, respectively, at three epidemiologically distinct sites in Myanmar. The aims were to determine whether low-density, subclinical malaria infections persisted over time and could be reliably detected using usPCR with successive testing, and whether certain demographic characteristics are associated with the prevalence and persistence of infection.

## MATERIALS AND METHODS

### Study design, sites, and populations.

**Figure 1.**
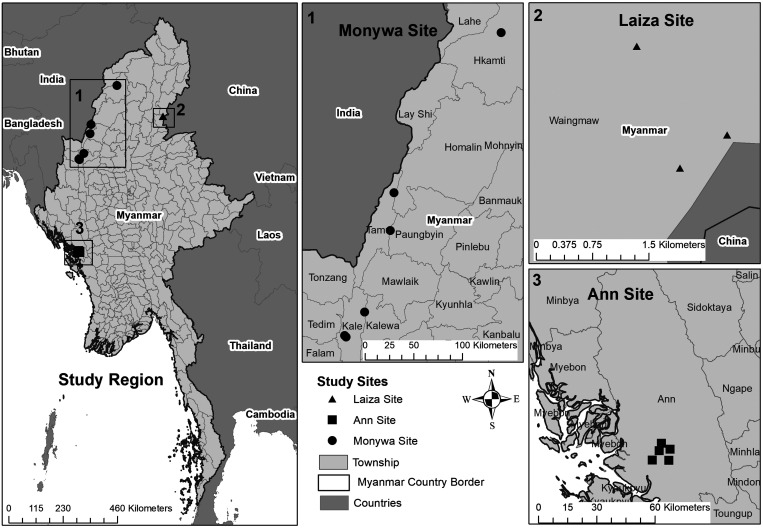
Map of study areas.

A prospective cohort study was performed to characterize short-term changes in subclinical infection status among participants living in three epidemiologically distinct regions of Myanmar. The study sites were chosen to represent a range of malaria epidemiologies characteristic of the region, including three military bases and three villages in Monywa Township in Myanmar’s Sagaing Region (referred to henceforth as “Monywa” site); two villages and one refugee community in Laiza Township in Kachin State, located on Myanmar’s border with China’s Yunnan Province (“Laiza” site); and five villages in Ann Township in Myanmar’s Rakhine State (“Ann” site) (Figure 1). Based on surveillance data and case reporting, Ann and Laiza Townships have been characterized as highly endemic with *P. falciparum* and *P. vivax* as the predominant species, respectively. Sagaing Region has historically had low malaria endemicity overall but with several “hot-spot” areas, including Monywa Township where both *P. falciparum* and *P. vivax* were suspected to be highly endemic. Selection of study villages or communities within each study site was based on known clinical malaria burden (unpublished data from annual reports of the Myanmar National Malaria Control Program and the National Institute of Parasitic Diseases, Chinese Center for Disease Control and Prevention), and on logistical considerations intended to ensure the integrity of study data and samples.

Two-step simple randomization was performed to select households within each study village or community, and participants within each household. A nearest next household was selected if originally selected household members were unavailable or declined to participate. Information about the study was disseminated within each study site by community engagement activities and by word-of-mouth, the most effective ways of research study recruitment in these remote communities, based on our previous experience. Inclusion criteria included age at least 6 months old, provision of a written informed consent or assent as appropriate, and ability and willingness to comply with the study protocol. Exclusion criteria included any clinical presentations that required immediate medical attention.

### Data collection.

After informed consent was obtained, demographic, medical and malaria history, behavioral, and travel-/mobility-related information were collected following a standardized questionnaire, using Open Data Kit (ODK) software, and uploaded to an Ona server and stored in a secure database at Duke University. Potential risk factors for malaria infection collected at baseline and during the follow-up period were age, gender, date of study data and sample collection (peak season is June–December, non–peak season is January–May), residence (defined as living in the study village for at least 6 months), type of occupation (dependent, student, soldier, refugee, farmer, plantation worker, or other, where “dependent” refers to a participant who did not work), setting of occupation (predominantly indoors versus predominantly outdoors), the presence of seasonal variation in primary occupation, and regular use of a bed net, defined as at least four+ nights per week. Travel patterns that were potentially related to malaria exposure were also investigated, including the distance from home to place of work or study, greatest distance traveled in a typical day, frequency of travel outside of the village in the last 6 months, mode of travel (nonmotorized or motorized vehicle), whether the participant stayed overnight at work, and frequency of exposure to known potential source of malaria, such as water source and forest.

The presence of clinical symptoms associated with malaria within the past 2 months and the past 24 hours was investigated, including headache, body ache, nausea, vomiting, abdominal discomfort, decreased appetite, fatigue, fever with chill and rigor, and fever. Oral/axillary temperature was taken and recorded at baseline and during follow-up visits, and fever was defined as temperature > 99.5°F. All study procedures were approved by institutional review boards at Duke University Medical Center (Protocol No. Pro00091895), Department of Medical Research, Myanmar, the Defense Services Medical Research Center, Myanmar and the National Institute of Parasitic Diseases, China CDC.

### Sample collection.

For the diagnosis of patent *P. falciparum* malaria infection, finger prick blood was tested by WHO-qualified RDT (Alere Malaria Ag P.f, Standard Diagnostics Bioline Inc., Gyeonggi-do, Republic of Korea) at baseline and any time during study follow-up as clinically indicated. Finger prick blood was spotted onto Whatman 903 and 3MM filter paper at baseline, during scheduled follow-up visits and unscheduled visits for ill symptoms, air-dried, and labeled with a unique participant identification number, initials of blood collector, and date and time of blood collection. These dried blood spot (DBS) samples were stored in individual sealed plastic bags with desiccant in a temperature-controlled facility until transported to central laboratories for molecular analysis.

### Study follow-up.

For participants who were RDT-positive at baseline, venous blood (up to 5 mL) was collected and stored in liquid nitrogen for future genotyping of malaria parasites. Referral was made, appropriate treatment by a malaria care provider following the national treatment guideline was ensured, and the treated participant was discharged from the study.

Participants who were RDT-negative and PCR-positive at visit #1 were followed for up to five additional visits (visit #2 to visit #6). In Laiza and Ann, the interval between visit #1 and visit #2 was 4 weeks, followed by biweekly interval for visits #3 to visit #6. In Monywa, the interval between visit #1 and visit #2 was 2 weeks, followed by 5 weekly follow-up visits. Participants who were RDT-negative and PCR-negative at baseline were tested again at visit #2, one week later in Monywa, and 2 weeks later in Laiza and Ann. Therefore, the total maximum number of observations for PCR-negative participants was two and for PCR-positive participants was six. The difference in the follow-up interval was due to logistical constraints for the military population at Monywa where lengthy follow-up (longer than 6 weeks) was not feasible.

### Laboratory methods.

From Whatman 3MM DBS samples, RNA and DNA were extracted using a β-mercaptoethanol-spiked guanidine thiocyanate-isopropanol buffer and purified using glass filter plates as previously described.^15^ Real-time reverse transcriptase usPCR analysis was performed following published methods targeting the 18S rRNA gene and transcripts for both *P. falciparum* and *P. vivax*, with an estimated LoD of 16 parasites/mL for *P. falciparum*.^5^^,^^15^^,^^16^ A β-tubulin internal control was amplified and detected for all samples to confirm successful DBS extraction. Samples were positive if duplicate measurements for a target had Cq values below 37. DBS samples collected in the Ann and Monywa study sites were analyzed in molecular laboratories located in Yangon and Naypyitaw, Myanmar, respectively, and those from Laiza were analyzed in a malaria laboratory in Shanghai, China, with technical support and coordination provided by a reference laboratory in the United States. All laboratories used identical Roche LightCycler 96 instruments and followed a defined standard operating procedure (SOP). Results were exported and processed using Microsoft Excel software before being merged into a final dataset together with the demographic and survey data. Consistency of methods across the participating laboratories and strict adherence to the SOPs were assured by prestudy training and ongoing refresher training provided by the reference laboratory. The usPCR results were reviewed in real time by experienced molecular analysts who ensured that all laboratory and process control criteria defined in the SOP were met. Subsets (10–20%) of samples from each country laboratory were selected randomly and shipped to the reference laboratory for independent quality control by a team blinded to initial results. Quality-assured usPCR results were shared in real time with the local study teams to guide follow-up activities.

### Statistical methods.

Statistics describing sociodemographic characteristics, malaria risk factors, and the history of clinical symptoms were computed and stratified by study site to characterize differences by geography. The sample baseline prevalence (henceforth referred to as “prevalence”) of *P. falciparum* and *P. vivax* were calculated by study site and subsite (by village or community), with exact binomial 95% CI, to visualize the heterogeneity in prevalence. Heterogeneity was quantified by the calculation of intraclass correlation coefficient (ICC) on village-level proportions using the analysis of variance (ANOVA) method.^17^ Baseline prevalence was also calculated by levels of categorical risk factors to aid interpretation of regression estimates.

Associations between sociodemographic characteristics, known malaria risk factors, and clinical symptom history and malaria positivity (*P. falciparum*, *P. vivax*, and all malaria) at screening were ascertained using individual modified Poisson regressions^18^ for each candidate variable. Exponentiated regression coefficients were interpreted as prevalence ratios (PRs). To account for sampling design, each regression was adjusted with indicator variables for study site. Each regression was also adjusted for potential confounding by age using dummy indicator variables representing age categories 0.5–16 years, 17–40 years, and 41+ years. Because the regression analyses were considered to be exploratory, no further adjustment for potential confounding was performed to reduce the risk of collider stratification bias and to interpret the regression parameters as age-adjusted total effects.

A similar approach was used to estimate factors associated with counts of positive malaria tests for those who had positive tests at baseline and during the follow-up period. Counts of positive tests did not distinguish between *P. falciparum* or *P. vivax*, but rather used baseline diagnosis of *P. falciparum*/*P. vivax* as a covariate to ascertain whether the species detected at baseline was associated with total counts of positive usPCR tests for malaria. To account for missing data during follow-up (e.g., missing samples or individuals who were either lost to follow-up after screening, had intermittent missing data, or who dropped out of the study after subsequent follow-up visits), a multiple imputation approach was used to fill in missing values that occurred at any point during follow-up and then to create a summary count of positives based on varying numbers of imputed values (i.e., super-varying variables). Covariates used to impute missing values for malaria diagnosis were data collection site, baseline indicators for *P. falciparum* and *P. vivax* (not mutually exclusive, owing to the occurrence of mixed infections with both species), age, occupation, hemoglobin result, distance to work/school, season occupation, occupation type (indoor *versus* outdoor), clinical symptoms (fever, chill, rigor, nausea, abdominal discomfort, headache, and body ache), female gender, regular use of mosquito net, measured body temperature, and season of data collection (rainy *versus* dry season).

Ten imputation datasets were produced for regression analysis, which used Poisson regression with robust standard errors and Rubin’s rules for combining results obtained from multiple imputation.^19^ Regression coefficients were exponentiated to give the interpretation of incident rate ratios (IRRs). Statistical inference focused on the magnitude and precision of PR and IRR estimates. Because of differences in sample populations and follow-up timing for Monywa, a sensitivity analysis was performed using data only from Laiza and Ann to assess consistency of findings for both baseline subclinical malaria positivity and counts of malaria positive tests over the follow-up time period. In the analysis of variables correlated with the count of positive tests over the 12-week period (or 6-week period for Monywa), indicator variables were added for species of *P. falciparum* and *P. vivax* (not mutually exclusive). Because this study was exploratory, no formal power calculation or statistical significance testing was performed.

## RESULTS

**Figure 2.**
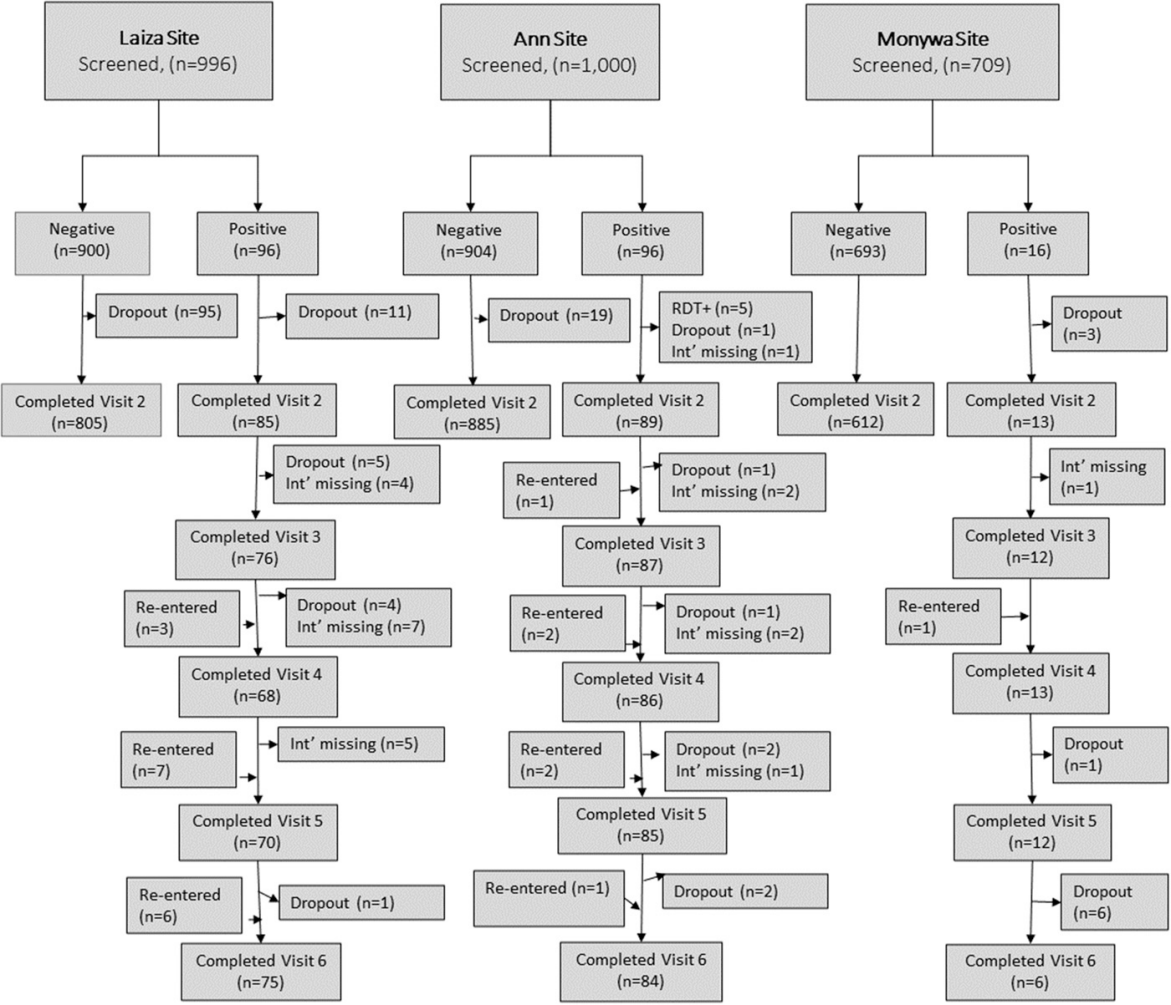
STROBE diagram for longitudinal sample flow, by study site and baseline malaria positivity.

The study was conducted between August 2018 and June 2019. The total number of participants enrolled at the Laiza, Ann, and Monywa study sites was 996, 1,000, and 709, respectively (Supplemental Figure 1). Three refugee villages or communities were enrolled at Laiza, with 240–480 participants enrolled per village or community. Five villages (80–345 per village) were enrolled at the Ann site, and six military communities (80–160 per community) were enrolled in Monywa. The number (%) of malaria-positive participants who remained in the study through the end of follow-up was 75 (78.1%), 84 (87.5%), and 6 (37.5%) in Laiza, Ann, and Monywa, respectively (Figure 2).

Table 1 summarizes demographic characteristics of the study population and their reported travel pattern. Participants in Laiza and Ann had similar age distributions, with a median age of 21 (interquartile range [IQR]: 11.0–38.5) and 23.5 years (IQR: 11.0, 41.0), respectively, whereas the median age in Monywa was slightly older at 33 years with narrower IQR of 26.0–46.0. A majority of participants was female in Ann and Laiza, while fewer than 5% of enrolled participants were female in Monywa. Laiza had a higher prevalence of students compared with the other sites, and Ann had a higher prevalence of farmers and plantation workers, while Monywa was composed primarily of military personnel (95.2%). Participants in Laiza reported limited travel for work, with at least 75% reporting less than 1 km of travel in a typical day, compared with participants in Ann and Monywa, where fewer than 50% reported limited travel.

**Table 1:** Characteristics of survey sample, by study site

Characteristic *N* (%) unless noted	Laiza site	Ann site	Monywa site	Total
	(*N* = 996)	(*N* = 1,000)	(*N* = 709)	(*N* = 2,705)
Age, median (IQR)	21.0 (11.0, 38.5)	23.5 (11.0, 41.0)	33.0 (26.0, 46.0)	27.0 (15.0, 42.0)
Age				
0.5–5	137 (13.8%)	100 (10.0%)	0 (0.0%)	237 (8.8%)
6–16	201 (20.2%)	286 (28.6%)	0 (0.0%)	487 (18.0%)
17–40	431 (43.3%)	363 (36.3%)	467 (65.9%)	1,261 (46.6%)
41–60	162 (16.3%)	186 (18.6%)	241 (34.0%)	589 (21.8%)
60+	65 (6.5%)	65 (6.5%)	1 (0.1%)	131 (4.8%)
Sex				
Male	388 (39.0%)	472 (47.2%)	676 (95.3%)	1,536 (56.8%)
Female	608 (61.0%)	528 (52.8%)	33 (4.7%)	1,169 (43.2%)
Type of main occupation				
Indoor	790 (79.3%)	526 (52.6%)	325 (45.8%)	1,641 (60.7%)
Outdoor	206 (20.7%)	474 (47.4%)	384 (54.2%)	1,064 (39.3%)
Main occupation				
Dependent	193 (19.4%)	240 (24.0%)	7 (1.0%)	440 (16.3%)
Student	466 (46.8%)	258 (25.8%)	2 (0.3%)	726 (26.8%)
Soldier	43 (4.3%)	1 (0.1%)	675 (95.2%)	719 (26.6%)
Refugee	146 (14.7%)	0 (0.0%)	0 (0.0%)	146 (5.4%)
Farmer	83 (8.3%)	141 (14.1%)	0 (0.0%)	224 (8.3%)
Plantation worker	5 (0.5%)	276 (27.6%)	0 (0.0%)	281 (10.4%)
Other	60 (6.0%)	84 (8.4%)	25 (3.5%)	169 (6.2%)
How far away is your place of work or study?				
< 1 km	841 (84.4%)	463 (46.3%)	457 (64.5%)	1,761 (65.1%)
1–5 km	128 (12.9%)	450 (45.0%)	114 (16.1%)	692 (25.6%)
> 5 km	27 (2.7%)	87 (8.7%)	138 (19.5%)	252 (9.3%)
What mode of travel do you use most frequently when you travel to work or study?				
Nonmotorized	586 (58.8%)	705 (70.5%)	549 (77.4%)	1,840 (68.0%)
Motorized	142 (14.3%)	43 (4.3%)	123 (17.3%)	308 (11.4%)
Work at home	268 (26.9%)	252 (25.2%)	37 (5.2%)	557 (20.6%)
Do you stay overnight for work or study?	189 (19.0%)	114 (11.4%)	459 (64.7%)	762 (28.2%)
Main occupation varied seasonally during the last year	6 (0.6%)	271 (27.1%)	36 (5.1%)	313 (11.6%)
How far is the greatest distance that you travel in a typical day?				
< 1 km	752 (75.5%)	343 (34.3%)	181 (25.5%)	1,276 (47.2%)
1–5 km	218 (21.9%)	562 (56.2%)	240 (33.9%)	1,020 (37.7%)
> 5 km	26 (2.6%)	95 (9.5%)	288 (40.6%)	409 (15.1%)
How many times have you traveled outside your village in the past 6 months?				
0–5 times	978 (99.5%)	924 (92.4%)	314 (44.4%)	2,216 (82.3%)
6+ times	5 (0.5%)	76 (7.6%)	394 (55.6%)	475 (17.7%)
Frequency of chores that involve trips to water				
Often (almost every day)	587 (58.9%)	859 (85.9%)	426 (60.1%)	1,872 (69.2%)
Rarely (special cases, e.g., burial)	409 (41.1%)	141 (14.1%)	283 (39.9%)	833 (30.8%)
Frequency of chores that involve trips to forest				
Often (almost every day)	409 (41.1%)	432 (43.2%)	295 (41.6%)	1,136 (42.0%)
Rarely (special cases, e.g., burial)	587 (58.9%)	568 (56.8%)	414 (58.4%)	1,569 (58.0%)
Do you use this mosquito net regularly (at least 4–5 nights per week)?	853 (85.6%)	868 (86.8%)	590 (83.2%)	2,311 (85.4%)
Lived in this village for > 6 months?	909 (91.3%)	994 (99.4%)	609 (85.9%)	2,512 (92.9%)

IQR = interquartile range.

### Baseline prevalence of malaria.

Table 2 reports the proportion of malaria at baseline by RDT (*P. falciparum* only) and usPCR (*P. falciparum* and *P.* vivax) by site. Of 2,705 enrolled, only 5 were positive for *P. falciparum* by RDT, all at the Ann site in Rakhine State. All RDT-positive participants also tested positive by usPCR. Using usPCR as a gold standard, the estimated specificity of the RDT for detecting *P. falciparum* was 100% (95% CI: 99.9–100.0%) with a sensitivity of the RDT of 2.4% (95% CI: 0.8–5.5%). By usPCR, malaria prevalence at baseline overall was similar (9.6%) in Laiza and Ann. All infections detected in Laiza were *P. vivax*, while *P. falciparum* prevalence was higher than *P. vivax* in Ann. Monywa had the lowest overall prevalence, with predominantly *P. vivax* infections. A small number (*N* = 7) of mixed *P. falciparum* and *P. vivax* infections was present only at the Ann site. Results of laboratory quality control procedures showed strong agreement between samples tested at site locations and Duke University. Specifically, at Department of Medical Research (DMR) (*N* = 96 samples), simple concordance was 96% (Cohen’s Kappa = 0.933) and at Defence Services Medical Research Centre (DSMRC) (*N* = 63), simple concordance was 95% (Cohen’s Kappa = 0.846).

**Table 2:** Test results and clinical characteristics, by study site

Characteristic, *N* (%) unless noted	Laiza site	Ann site	Monywa site	Total
	(*N* = 996)	(*N* = 1,000)	(*N* = 709)	(*N* = 2,705)
usPCR result				
Negative	900 (90.4%)	904 (90.4%)	693 (97.7%)	2,497 (92.3%)
* P. falciparum*	0 (0.0%)	38 (3.8%)	2 (0.3%)	40 (1.5%)
* P. falciparum* + *P. vivax*	0 (0.0%)	7 (0.7%)	0 (0.0%)	7 (0.3%)
* P. vivax*	96 (9.6%)	51 (5.1%)	14 (2.0%)	161 (6.0%)
Conventional Rapid Diagnostic Test result				
Negative	996 (100.0%)	995 (99.5%)	709 (100.0%)	2,700 (99.8%)
P. falciparum	0 (0.0%)	5 (0.5%)	0 (0.0%)	5 (0.2%)
Self-reported symptoms (past 2 months)				
Headache	496 (50.2%)	570 (57.1%)	285 (40.2%)	1,351 (50.1%)
Body ache/pain	345 (34.8%)	483 (48.4%)	358 (50.5%)	1,186 (44.0%)
Nausea	150 (15.2%)	67 (6.7%)	63 (8.9%)	280 (10.4%)
Vomiting	136 (13.7%)	71 (7.1%)	55 (7.8%)	262 (9.7%)
Abdominal discomfort	298 (29.9%)	314 (31.4%)	173 (24.4%)	785 (29.0%)
Decreased appetite	305 (30.6%)	189 (18.9%)	134 (18.9%)	628 (23.2%)
Fatigue	253 (25.5%)	157 (15.7%)	130 (18.3%)	540 (20.0%)
Fever with chill and rigor	142 (14.3%)	83 (8.3%)	100 (14.1%)	325 (12.0%)
Self-reported symptoms (past 24 hours)				
In the past 24 hours, have you had a fever?	52 (5.2%)	16 (1.6%)	28 (3.9%)	96 (3.6%)
Measured clinical characteristics				
Body temperature (°F), median (IQR)	97.9 (97.5, 98.4)	97.7 (97.3, 98.2)	97.9 (97.2, 98.4)	97.9 (97.3, 98.4)
Febrile (body temperature ≥ 99.5°F)	16 (1.6%)	38 (3.8%)	13 (1.8%)	67 (2.5%)
Hemoglobin level (g/dL), median (IQR)	12.8 (11.8, 13.8)	12.5 (11.6, 13.5)	14.2 (13.2, 15.3)	13.0 (12.0, 14.2)

IQR = interquartile range; usPCR = ultrasensitive polymerase chain reaction.

**Figure 3.**
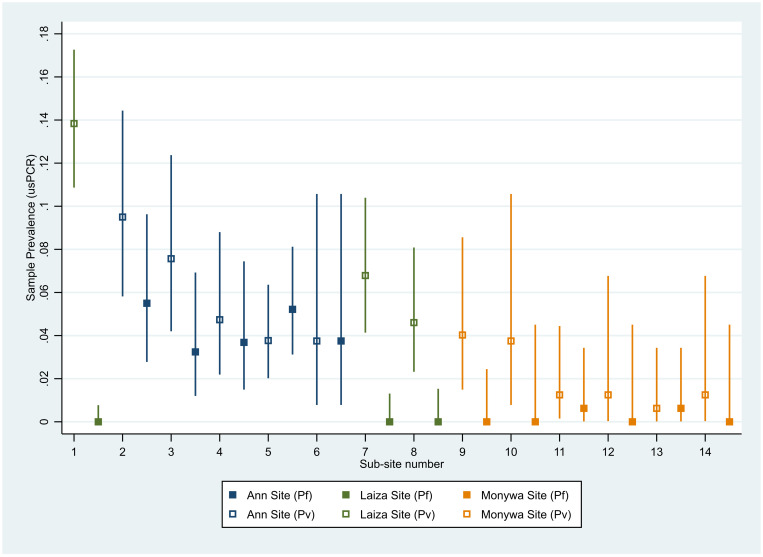
*Plasmodium falciparum* and *P. vivax* baseline sample prevalence with 95% CIs, by study site subarea. This figure appears in color at www.ajtmh.org.

Within each of the three study sites, usPCR-positive malaria prevalence varied among villages or communities. The range of prevalence in Laiza was 4.6% (95% CI 2.3–8.1) to 13.8% (95% CI 10.8–17.3) (Figure 3, Supplemental Table 2). In Ann, the range was 7.5% (95% CI 2.8–15.6) to 13.0% (95% CI 8.7–18.5). In Monywa, prevalence ranged from 1.3% (95% CI 0.03–6.7) to 4.0% (95% CI 1.5–8.6). To assess heterogeneity in village-level prevalence of *P. falciparum*, *P. vivax*, and mixed *P. falciparum/P. vivax* infections, ICC estimates were compared between the three different infection states. The highest between-village heterogeneity was observed for *P. vivax* (ICC = 0.028, 95% CI 0.001, 0.055) and the lowest was observed for all malaria (ICC = 0.023, 95% CI: 0.000, 0.046).

### Temporal dynamics of malaria infection.

Of the 2,705 enrolled, 2,497 participants (92.3%) tested negative by usPCR at baseline (visit #1), with 2,249 (90.1%) remaining PCR-negative at follow-up visit #2. Fifty-three (2.1%) who tested negative at visit #1 were positive by usPCR at visit #2, and 195 (7.8%) who were negative at visit #1 were lost to follow-up at visit #2.

**Figure 4.**
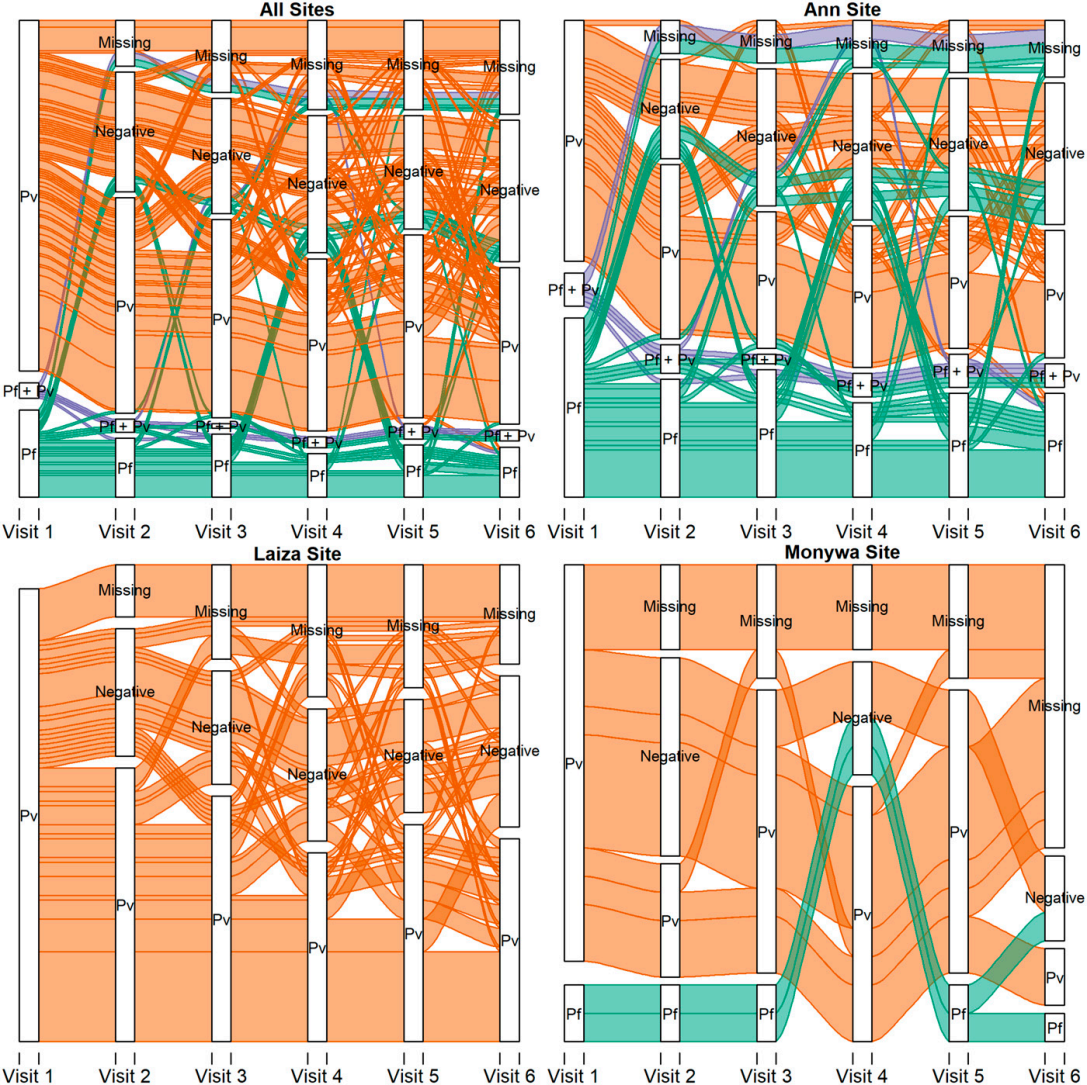
Trajectories of diagnosis by visit (alluvial), by study site. This figure appears in color at www.ajtmh.org.

Of the 208 participants who were positive by usPCR at baseline, data for all six study visits were available for 146 participants (70.2%). In Laiza and Ann, 72.4% of *P. falciparum* or *P. vivax* positive at visit #1 were still positive at visit #2 one month later, and 37.1% of *P. falciparum* or *P. vivax* positive participants remained positive at all six study visits (Figure 4). In Monywa, 46% of PCR-positive at visit #1 remained positive at visit #2, 2 weeks later, whereas 33.3% were positive at all six weekly follow-up visits.

For the 146 participants who were positive by usPCR at baseline, the patterns of positive and negative tests were relatively equally split between participants who remained PCR-positive for all study visits (*N* = 54, 37.0%), participants who tested negative at least once and subsequently remained negative (*N* = 45, 30.8%), and those who were intermittently positive and negative (*N* = 47, 32.2%) (Supplemental Table 3). Persistence of infection at all six study visits was found in a higher number of participants at the Ann site (*N* = 33, 41.8%) than the other sites, and those whose initial PCR results were positive for *P. falciparum* tended to remain positive throughout the study (*N* = 17, 48.6%) compared with those who tested positive for *P. vivax* (*N* = 40, 35.1%). No differences were seen in the proportion of participants at the different sites who were intermittently positive, comparing baseline *P. falciparum* and *P. vivax* infections.

### Risk factors of subclinical infection.

At baseline, malaria infections were most common among refugees (15.8%), plantation workers (14.6%), and farmers (11.2%) and tended be more common among those with outdoor occupations (10.6% versus 5.8% for indoor occupations) (Supplemental Table 4). Those with symptoms suggestive of malaria both at enrollment and over the previous 2 months had slightly higher rates of baseline malaria infection compared with those who did not have symptoms. Likewise, those reporting more frequent trips to forest and water saw higher baseline prevalence of malaria compared with those reporting frequency of chores as rare or never. At baseline, 3.9% of “dependents” had subclinical infection, among the lowest malaria prevalence of all occupation categories. Plantation workers had the highest relative difference in prevalence of subclinical infection for any occupation category compared with dependents (PR = 4.09, 95% CI 2.23, 7.51), after adjustment for site and age (Table 3). Among all nonreferent occupation categories, students had moderately higher rates of positive malaria tests over the follow-up time period, compared with dependents (IRR = 1.46, 95% CI 1.08, 1.97). Those with occupations classified as occurring predominantly outdoors were more likely to test positive for malaria at baseline, with unadjusted baseline prevalence for outdoor occupation of 10.6% *versus* 5.8% for those with indoor occupations (PR = 2.57, 95% CI 1.84, 3.58); however, those with outdoor occupations also saw fewer subsequent positive tests (IRR = 0.66, 95% CI 0.55, 0.80) than individuals with indoor occupations who tested positive at baseline.

**Table 3:** Results of regression models assessing correlates of baseline subclinical malaria infection and rate of repeated subclinical infection status over time, complete case, and multiple imputed

	Baseline prevalence *N* = 2,705		Count of positives (complete case)* N* = 146		Count of positives (multiple imputation)* N* = 208	
Independent variable	Prevalence ratio (95% CI)	*P* value	Incident rate ratio (IRR) (95% CI)	*P* value	Incident rate ratio (IRR) (95% CI)	*P* value
Age, *n* (%)						
0.5–16	(ref)	–	(ref)	–	(ref)	–
17–40	1.31 (0.95, 1.82)	0.103	0.59 (0.37, 0.95)	0.029	0.80 (0.65, 0.99)	0.045
41+	1.59 (1.13, 2.25)	0.009	0.57 (0.36, 0.90)	0.017	0.74 (0.59, 0.91)	0.006
Sex						
Male	(ref)	–	(ref)	–	(ref)	–
Female	0.62 (0.47, 0.82)	0.001	1.00 (0.72, 1.40)	0.981	1.02 (0.86, 1.21)	0.822
Type of main occupation						
Indoor	(ref)	–	(ref)	–	(ref)	–
Outdoor	2.57 (1.84, 3.58)	< 0.001	0.75 (0.47, 1.18)	0.216	0.66 (0.55, 0.80)	< 0.001
What is your main occupation?						
Dependent	(ref)	–	(ref)	–	(ref)	–
Student	2.19 (1.29, 3.73)	0.004	1.25 (0.57, 2.74)	0.582	1.46 (1.08, 1.97)	0.015
Soldier	3.88 (1.69, 8.93)	0.001	0.93 (0.27, 3.23)	0.914	0.88 (0.57, 1.37)	0.573
Refugee	3.86 (1.94, 7.68)	< 0.001	0.73 (0.32, 1.67)	0.462	0.87 (0.67, 1.13)	0.309
Farmer	2.92 (1.54, 5.54)	0.001	0.96 (0.39, 2.38)	0.933	0.99 (0.74, 1.32)	0.943
Plantation worker	4.09 (2.23, 7.51)	< 0.001	0.88 (0.36, 2.18)	0.783	0.94 (0.69, 1.29)	0.707
Other	3.36 (1.73, 6.51)	< 0.001	1.00 (0.39, 2.60)	0.995	1.10 (0.76, 1.61)	0.605
How far away is your place of work or study?						
< 1 km	(ref)	–	(ref)	–	(ref)	–
1–5 km	1.93 (1.43, 2.59)	< 0.001	1.06 (0.72, 1.57)	0.764	1.00 (0.77, 1.30)	0.992
> 5 km	2.40 (1.57, 3.65)	< 0.001	1.07 (0.60, 1.93)	0.815	0.97 (0.77, 1.22)	0.799
Most frequent mode of travel to work/study						
Nonmotorized	(ref)	–	(ref)	–	(ref)	–
Motorized	1.13 (0.76, 1.67)	0.548	0.62 (0.39, 0.99)	0.046	0.69 (0.54, 0.87)	0.003
Work at home	0.39 (0.26, 0.60)	< 0.001	1.13 (0.63, 2.03)	0.678	1.02 (0.84, 1.24)	0.806
Do you stay overnight for work or study?						
No	(ref)	–	(ref)	–	(ref)	–
Yes	1.24 (0.89, 1.72)	0.212	0.97 (0.66, 1.42)	0.857	1.02 (0.85, 1.23)	0.803
Did your main occupation vary seasonally in the past 1 year?						
No	(ref)	–	(ref)	–	(ref)	–
Yes	1.37 (0.93, 2.03)	0.111	0.84 (0.51, 1.37)	0.484	0.82 (0.67, 1.01)	0.067
How far is the greatest distance that you travel in a typical day?						
< 1 km	(ref)	–	(ref)	–	(ref)	–
1–5 km	1.83 (1.35, 2.50)	< 0.001	1.11 (0.76, 1.64)	0.585	0.95 (0.75, 1.19)	0.622
> 5 km	2.60 (1.67, 4.04)	< 0.001	1.03 (0.61, 1.75)	0.906	0.87 (0.72, 1.06)	0.169
How many times have you traveled outside your village in the past 6 months?						
0–5 times	(ref)	–	(ref)	–	(ref)	–
6+ times	0.81 (0.46, 1.42)	0.456	0.97 (0.48, 1.94)	0.931	1.09 (0.83, 1.43)	0.523
Frequency of chores that involve trips to water						
Often (almost every day)	(ref)	–	(ref)	–	(ref)	–
Rarely (special cases, e.g., burial)	0.50 (0.35, 0.72)	< 0.001	0.81 (0.55, 1.18)	0.274	0.93 (0.80, 1.08)	0.327
Frequency of chores that involve trips to forest						
Often (almost every day)	(ref)	–	(ref)	–	(ref)	–
Rarely (special cases, e.g., burial)	0.54 (0.41, 0.72)	< 0.001	1.23 (0.86, 1.77)	0.255	1.23 (1.05, 1.44)	0.013
Do you use this mosquito net regularly (at least 4–5 nights per week)?						
No	(ref)	–	(ref)	–	(ref)	–
Yes	0.74 (0.53, 1.05)	0.089	1.26 (0.80, 1.98)	0.326	1.20 (1.01, 1.43)	0.035
Residence (Have you lived in this village for > 6 months?)						
No	(ref)	–	(ref)	–	(ref)	–
Yes	1.44 (0.71, 2.89)	0.308	1.15 (0.58, 2.25)	0.688	1.13 (0.85, 1.51)	0.400
Timing of baseline data collection						
Peak (June–December)	(ref)	–	(ref)	–	(ref)	–
Nonpeak (January–May)	1.09 (0.83, 1.43)	0.534	0.88 (0.60, 1.30)	0.526	0.92 (0.79, 1.08)	0.318
usPCR result						
* P. vivax* positive at baseline	–	–	0.67 (0.41, 1.08)	0.102	0.77 (0.63, 0.94)	0.010
* P. falciparum* positive at baseline	–	–	1.68 (1.04, 2.73)	0.035	1.34 (1.11, 1.63)	0.003
Self-reported symptoms (past 2 months)						
Headache	1.08 (0.82, 1.43)	0.569	1.25 (0.90, 1.73)	0.188	1.13 (0.96, 1.34)	0.132
Body ache/pain	1.27 (0.94, 1.71)	0.118	1.29 (0.93, 1.80)	0.131	1.29 (1.14, 1.46)	< 0.001
Nausea	1.24 (0.84, 1.83)	0.284	1.22 (0.73, 2.04)	0.441	1.19 (0.95, 1.49)	0.126
Vomiting	1.31 (0.89, 1.94)	0.173	1.38 (0.79, 2.42)	0.260	1.44 (1.04, 1.99)	0.030
Abdominal discomfort	0.98 (0.73, 1.30)	0.863	1.05 (0.73, 1.52)	0.787	1.11 (0.96, 1.29)	0.146
Decreased appetite	0.95 (0.70, 1.28)	0.732	1.02 (0.69, 1.51)	0.930	1.08 (0.93, 1.25)	0.318
Fatigue	1.06 (0.77, 1.45)	0.731	0.81 (0.57, 1.17)	0.262	0.92 (0.77, 1.10)	0.356
Fever with chill and rigor	1.25 (0.86, 1.83)	0.242	1.29 (0.73, 2.29)	0.387	1.26 (0.95, 1.66)	0.100
Self-reported symptoms (past 24 hours)						
In the past 24 hours, have you had a fever?	1.11 (0.57, 2.17)	0.760	0.88 (0.37, 2.10)	0.773	1.06 (0.75, 1.51)	0.729
Measured clinical characteristics						
Temperature (°F) (standardized)	1.09 (0.95, 1.25)	0.204	1.11 (0.91, 1.34)	0.300	1.06 (0.99, 1.13)	0.110
Febrile (body temperature ≥ 99.5°F)	1.44 (0.74, 2.81)	0.278	1.14 (0.48, 2.69)	0.763	1.25 (0.86, 1.84)	0.237
Hemoglobin (standardized)	1.11 (0.95, 1.30)	0.193	0.99 (0.83, 1.18)	0.907	1.01 (0.94, 1.09)	0.737

IRR = incident rate ratios; usPCR = ultrasensitive polymerase chain reaction.

Older age groups were more likely than children aged 6 months to 16 years to have subclinical malaria at baseline (PR = 1.59, 95% CI 1.13, 2.25), although the older age groups had fewer positive counts over the follow-up time points than younger children who had tested positive at baseline (IRR = 0.80, 95% CI 0.65, 0.99 for individuals aged 17–40 years, IRR = 0.74, 95% CI: 0.59, 0.91 for those aged 41 years or older).

Those who traveled farther in a typical day were more likely to have subclinical malaria at baseline, with rates of positivity increasing with increasing distance traveled (PR = 1.83, 95% CI 1.35, 2.50 for those traveling 1–5 km; PR = 2.60, 95% CI 1.67, 4.04 for those traveling > 5 km) although travel history did not correlate with the subsequent number of positive tests.

Individuals who performed chores involving frequent trips to water sources and forests were more likely to test positive for malaria at baseline (PR = 2.00, 95% CI 1.39, 2.86 for trips to water; PR = 1.85, 95% CI 1.39, 2.43 for trips to forest). Subsequent counts of positive tests were modestly correlated with lower frequency of trips to forest (IRR = 1.23, 95% CI 1.05, 1.44) but not strongly correlated with lower frequencies of trips to water.

The time of year during which screening occurred did not correlate well with either the probability of being positive at baseline (PR = 1.09, 95% CI 0.83, 1.43 for screening during non–peak season *versus* peak season reference) or the subsequent number of positive tests for those who tested positive at baseline (IRR = 0.92, 95% CI 0.79, 1.08).

Individuals who tested positive for *P. falciparum* at baseline tended to have higher subsequent counts of positives than those who tested positive only for *P. vivax* (IRR = 1.34, 95% CI 1.11, 1.63). Self-reported symptoms suggestive of malaria in the 2 months preceding screening were not correlated with the probability of testing positive at baseline, although reports of body ache/pain (IRR = 1.29, 95% CI 1.14, 1.46) and vomiting (IRR = 1.44, 95% CI 1.04, 1.99) were associated with higher counts of positives tests for those testing positive at baseline.

Results remained largely unchanged in a sensitivity analysis when data from Monywa were excluded (Supplemental Table 5), with only modest changes in estimated magnitudes of association between measured temperature and subclinical malaria at baseline and the counts of subsequent positive malaria results for individuals reporting abdominal discomfort in the 2 months preceding baseline screening.

Overall, those potential risk factors that were associated with testing positive for subclinical malaria at baseline were not associated with the count of positives that occurred after baseline. In several cases, factors predicting initial infection produced effects on counts of positives that were in the opposite direction (e.g., age, trips to forest, and indoor/outdoor occupation type).

## DISCUSSION

Results from this study add to the growing body of evidence supporting the existence of a silent reservoir of subclinical malaria infections in the Greater Mekong Subregion that may pose a challenge to the continued progress toward malaria elimination in the region. The finding that almost all infections were both clinically silent (subclinical) and undetected by standard testing (subpatent) suggests that traditional surveillance methods based on rapid diagnostic testing may be inadequate to detect residual malaria infections in these settings. It should be noted that screening was intentionally targeted to potential high-risk groups and communities; therefore, prevalence estimates may not be representative of the underlying target populations. Even so, the estimated prevalences were similar to those found in previous studies in the region^7^^,^^13^^,^^20^^–^^22^ and further support high heterogeneity in the prevalence of subclinical infection at the community level. Somewhat surprisingly, the prevalence of subclinical malaria among military personnel in Monywa was much lower on average than among civilian participants, despite their presumed exposure to risk factors such as forests and outdoor activities. This finding, if confirmed, would suggest that similar military populations, despite their frequent travel across Myanmar, may not be as large a threat to geographic spread of malaria parasites as had been presumed. A larger representative sample of military personnel would need to be studied to confirm this finding.

Although parasite densities cannot be derived from the results of our nonquantitative PCR assay, the short-term temporal dynamics of subclinical malaria infection observed in this study suggest that both *P. falciparum* and *P. vivax* parasite densities can vary substantially over the course of 6–12 weeks. The PCR test used for this study has lower limits of detection of approximately 16 parasites/mL for *P. falciparum* and 49.5 copies/μL for *P. vivax*,^5^ more than 200-fold more sensitive than conventional PCR tests. In these low transmission settings, it is likely that the overwhelming majority of changes in infection status from negative to positive (or positive to negative) represent fluctuations above and below these very low thresholds of detection, and not serial cure and reinfection over the study period, although this possibility cannot be eliminated.

These patterns of intermittent PCR-positivity suggest that even usPCR may miss a substantial number of infections if only a single test is done to ascertain infection status. Less-sensitive laboratory-based and point-of-contact molecular and flow-based detection methods will miss even more infections that fluctuate below the higher detection thresholds for these tests. As expected, participants with *P. vivax* infections had a lower rate of incident positive test results overall, which is likely related both to the parasite’s ability to go undetected during its latent liver stage infection, and to the comparatively lower sensitivity of the *P. vivax* usPCR. This suggests that even more *P. vivax* infections may go undetected when only a single test is performed. In this short-term cohort study, it was not possible to identify when new infections originated and when they permanently disappeared.

Mass (or focal) screening and treatment strategies involve testing all people in a given area or population, and treating only those who test positive. The regression analyses in this study support previous work showing that in this region, targeting certain groups based on older age, male gender, high-risk occupation, and frequent engagement in other outdoor activities, like collecting water and time spent in the forest, may improve the effectiveness of these screen-and-treat strategies. The high heterogeneity seen in prevalence of subclinical malaria even in adjacent villages is consistent with previous research,^14^ and suggests that elimination efforts should target specific foci within geographical units such as at the township level.

In this observational study, unmeasured confounding cannot be ruled out as a possible explanation for some findings. Furthermore, measurement error due to the imperfect sensitivity of our PCR test may have led to bias in PR estimates, although this bias would be small unless the proportion of participants who had baseline parasite densities below the lower limit of detection (i.e., false negatives) was different across exposure strata. Finally, if false positives occurred (e.g., as a result of residual plasmodial genetic material from resolved infections circulating in the blood at the time of sample collection), prevalence estimates could have been falsely elevated. The predominant pattern of repeated positive results suggests that any such false positives likely occurred rarely.

The probability of continuous infection over time was lower in older people and those exposed to outdoor environments, the same factors found to increase prevalence of infection at baseline screening. It is possible that these groups are able to clear infection more rapidly because of higher levels of immunity acquired through repeated exposures. Irrespective of demographic characteristics, no individuals who had subclinical infection developed symptomatic malaria illness over the 6- or 12-week follow-up period. Again, additional cohort studies with longer follow-up will be needed to assess whether certain groups are able to resolve infections more quickly, and whether and how often these subclinical infections become symptomatic.

Importantly, the lack of any differences in either the prevalence or count of subclinical infections by season supports the possibility that transmission potential (in the form of persistent subclinical infections) could be maintained throughout the dry season in these low endemicity settings, raising the prospect of malaria elimination interventions that target this “silent” reservoir when transmission is not occurring.^21^^–^^23^

Despite the dramatic progress toward eliminating malaria from the Greater Mekong Subregion, this study confirms that even in areas with very few reported cases of malaria illness, a reservoir of subclinical infection persists year-round. If these low-density infections are shown to represent a significant source of transmission, identification of high-risk groups and locations (“hot pops” and “hot spots”) may aid elimination efforts. However, identifying these populations will be difficult with available technologies, given the dynamic nature of *P. falciparum* and *P. vivax* densities over time and the lack of highly sensitive tests that can be performed in the field. With the ability to detect evidence of recent past infection, serological testing may offer a more promising approach for surveillance in these elimination settings if reliable point-of-contact tests can be developed and validated.^24^^,^^25^

## Supplemental files


Supplemental materials
