# Supplementary material for: Temporal Dynamics of Subclinical Malaria in Different Transmission Zones of Myanmar
Source: Am J Trop Med Hyg. 2022 Jul 25;107(3):669–80. doi: 10.4269/ajtmh.22-0027 (PMC9490656; doi:10.4269/ajtmh.22-0027)
Supplement: Supplementary file 1 [file tpmd220027.SD1.pdf]

Supplemental Figure 1. Data collection over time, by study site.

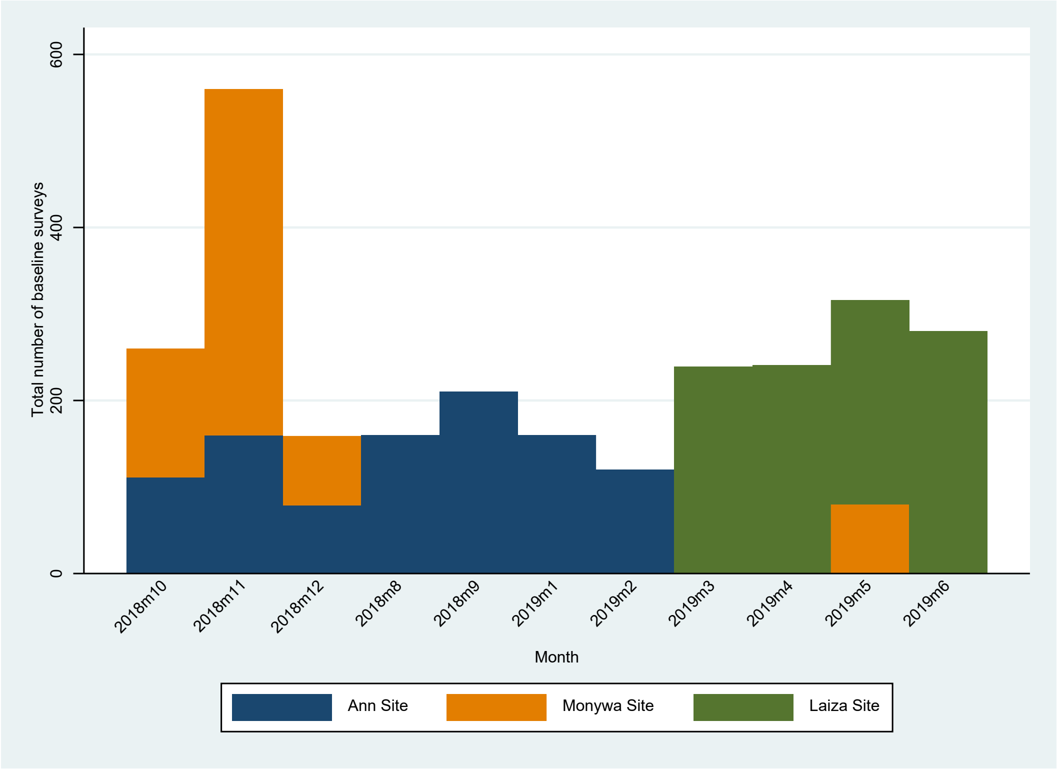

Supplemental Table 1. Expected versus actual person time follow-up

Site	Expected Person Visits[1]	Actual Person Visits[2]	% Person Visits Achieved
**Baseline Negative**			
Laiza Site	1800	1705	94.7
Ann Site	1808	1789	98.9
Monywa Site	1386	1305	94.2
Total	4994	4799	96.1
**Baseline Positive**			
Laiza Site	576	470	81.6
Ann Site	551	527	95.6
Monywa Site	96	72	75.0
Total	1223	1069	87.4

[1]Expected person visits incorporates sampling plan of 2 visits (for baseline negatives) and 6 visits (for baseline positives), and 1 visit for those testing positive by RDT at baseline

[2]Actual person visits tabulates the actual number of study visits where a participant was present, stratified by baseline positivity

Supplemental Table 2. Sample proportions of *P. falciparum*, *P. vivax*, and mixed infections by area

Area Name	Obs	All Malaria: n, proportion (95% CI)	P. falciparum: n, proportion (95% CI)	P. vivax: n, proportion (95% CI)	Mixed Infection: n, proportion (95% CI)
All	2705	208, 7.69 (6.71,8.76)	47, 1.74 (1.28,2.30)	168, 6.21 (5.33,7.19)	7, 0.26 (0.10,0.53)
Laiza Site	996	96, 9.64 (7.88,11.64)	0, 0.00 (0.00,0.37)	96, 9.64 (7.88,11.64)	0, 0.00 (0.00,0.37)
Ann Site	1000	96, 9.60 (7.85,11.60)	45, 4.50 (3.30,5.98)	58, 5.80 (4.43,7.43)	7, 0.70 (0.28,1.44)
Monywa Site	709	16, 2.26 (1.30,3.64)	2, 0.28 (0.03,1.02)	14, 1.97 (1.08,3.29)	0, 0.00 (0.00,0.52)
**Laiza Site**	.				
Sub-site 1	477	66, 13.84 (10.87,17.26)	0, 0.00 (0.00,0.77)	66, 13.84 (10.87,17.26)	0, 0.00 (0.00,0.77)
Sub-site 8	239	11, 4.60 (2.32,8.09)	0, 0.00 (0.00,1.53)	11, 4.60 (2.32,8.09)	0, 0.00 (0.00,1.53)
Sub-site 7	280	19, 6.79 (4.13,10.39)	0, 0.00 (0.00,1.31)	19, 6.79 (4.13,10.39)	0, 0.00 (0.00,1.31)
**Ann Site**	.				
Sub-site 5	345	29, 8.41 (5.70,11.85)	18, 5.22 (3.12,8.12)	13, 3.77 (2.02,6.36)	2, 0.58 (0.07,2.08)
Sub-site 4	190	16, 8.42 (4.89,13.32)	7, 3.68 (1.49,7.44)	9, 4.74 (2.19,8.80)	0, 0.00 (0.00,1.92)
Sub-site 3	185	19, 10.27 (6.30,15.57)	6, 3.24 (1.20,6.93)	14, 7.57 (4.20,12.37)	1, 0.54 (0.01,2.97)
Sub-site 2	200	26, 13.00 (8.67,18.47)	11, 5.50 (2.78,9.63)	19, 9.50 (5.82,14.44)	4, 2.00 (0.55,5.04)
Sub-site 6	80	6, 7.50 (2.80,15.61)	3, 3.75 (0.78,10.57)	3, 3.75 (0.78,10.57)	0, 0.00 (0.00,4.51)
**Monywa Site**	.				
Sub-site 9	149	6, 4.03 (1.49,8.56)	0, 0.00 (0.00,2.45)	6, 4.03 (1.49,8.56)	0, 0.00 (0.00,2.45)
Sub-site 14	160	2, 1.25 (0.15,4.44)	1, 0.63 (0.02,3.43)	1, 0.63 (0.02,3.43)	0, 0.00 (0.00,2.28)
Sub-site 11	160	3, 1.88 (0.39,5.38)	1, 0.63 (0.02,3.43)	2, 1.25 (0.15,4.44)	0, 0.00 (0.00,2.28)
Sub-site 10	80	3, 3.75 (0.78,10.57)	0, 0.00 (0.00,4.51)	3, 3.75 (0.78,10.57)	0, 0.00 (0.00,4.51)
Sub-site 13	80	1, 1.25 (0.03,6.77)	0, 0.00 (0.00,4.51)	1, 1.25 (0.03,6.77)	0, 0.00 (0.00,4.51)
Sub-site 12	80	1, 1.25 (0.03,6.77)	0, 0.00 (0.00,4.51)	1, 1.25 (0.03,6.77)	0, 0.00 (0.00,4.51)
Intraclass Correlation Coefficient	2709	0.023 (0.000,0.046)	0.026 (0.000,0.052)	0.028 (0.001,0.055)	

Supplemental Table 3. usPCR positivity patterns over all follow-up visits for individuals testing malaria positive at baseline and providing data for all study visits, stratified by site, species

	Study Site			Baseline Species				
				P. falciparum		*P. vivax*		
	Ann Site	Monywa Site	Laiza Site	Negative	Positive	Negative	Positive	Total
	(N = 79)	(N = 6)	(N = 61)	(N = 111)	(N = 35)	(N = 32)	(N = 114)	(N = 146)
**Positivity Pattern**								
Always positive	33 (41.8%)	2 (33.3%)	19 (31.1%)	37 (33.3%)	17 (48.6%)	14 (43.8%)	40 (35.1%)	54 (37.0%)
Monotonic positive to negative	22 (27.8%)	0 (0.0%)	23 (37.7%)	38 (34.2%)	7 (20.0%)	7 (21.9%)	38 (33.3%)	45 (30.8%)
Intermittent positive	24 (30.4%)	4 (66.7%)	19 (31.1%)	36 (32.4%)	11 (31.4%)	11 (34.4%)	36 (31.6%)	47 (32.2%)

Supplemental Table 4. Sample prevalence of *P. falciparum*, *P. vivax*, and all malaria by levels of categorical risk factors

Risk factor	P. falciparum	P. vivax	All malaria
	Prevalence (95% CI)	Prevalence (95% CI)	Prevalence (95% CI)
Age			
0.5-16	1.9 (1.1,3.2)	6.4 (4.7,8.4)	7.6 (5.8,9.8)
17-40	1.5 (0.9,2.3)	5.7 (4.5,7.1)	7.1 (5.7,8.6)
41+	1.9 (1.1,3.2)	6.9 (5.2,9.1)	8.9 (6.9,11.2)
Sex			
Male	1.6 (1.0,2.3)	6.4 (5.3,7.8)	7.7 (6.5,9.2)
Female	2.0 (1.3,2.9)	5.9 (4.6,7.4)	7.6 (6.2,9.3)
Type of main occupation			
Indoor	1.0 (0.6,1.6)	5.1 (4.0,6.2)	5.8 (4.7,7.0)
Outdoor	2.9 (2.0,4.1)	8.0 (6.4,9.8)	10.6 (8.8,12.6)
What is your main occupation?			
Dependent	0.9 (0.2,2.3)	3.0 (1.6,5.0)	3.9 (2.3,6.1)
Student	1.5 (0.8,2.7)	7.6 (5.8,9.7)	8.5 (6.6,10.8)
Soldier	0.3 (0.0,1.0)	2.8 (1.7,4.3)	3.1 (1.9,4.6)
Refugee	0.0 (0.0,2.5)	15.8 (10.3,22.7)	15.8 (10.3,22.7)
Farmer	2.2 (0.7,5.1)	8.9 (5.5,13.5)	11.2 (7.4,16.0)
Plantation worker	7.1 (4.4,10.8)	8.2 (5.3,12.0)	14.6 (10.7,19.3)
Other	3.0 (1.0,6.8)	8.3 (4.6,13.5)	10.7 (6.4,16.3)
How far away is your place of work or study?			
<1 km	0.6 (0.3,1.1)	5.4 (4.4,6.6)	6.0 (4.9,7.2)
1-5 km	3.8 (2.5,5.5)	8.2 (6.3,10.5)	11.3 (9.0,13.9)
>5 km	4.0 (1.9,7.2)	6.3 (3.7,10.1)	9.9 (6.5,14.3)
What mode of travel do you use most frequently when you travel to work or s			
Non-motorized	2.2 (1.6,3.0)	6.6 (5.5,7.9)	8.5 (7.3,9.9)
Motorized	0.6 (0.1,2.3)	8.4 (5.6,12.1)	8.8 (5.9,12.5)
Work at home	0.7 (0.2,1.8)	3.6 (2.2,5.5)	4.3 (2.8,6.3)
Do you stay overnight for work or study?			
No	1.6 (1.1,2.3)	6.8 (5.7,8.0)	8.2 (7.1,9.5)
Yes	2.0 (1.1,3.2)	4.7 (3.3,6.5)	6.3 (4.7,8.3)
Does your main occupation vary seasonally in the past one year?			
No	1.2 (0.8,1.7)	6.2 (5.3,7.2)	7.1 (6.1,8.3)
Yes	5.8 (3.4,8.9)	6.4 (3.9,9.7)	11.8 (8.5,15.9)
How far is the greatest distance that you travel in a typical day?			
<1 km	0.7 (0.3,1.3)	5.6 (4.4,7.1)	6.3 (5.0,7.7)
1-5 km	2.6 (1.8,3.8)	7.5 (5.9,9.2)	9.6 (7.9,11.6)
>5 km	2.7 (1.4,4.8)	4.9 (3.0,7.5)	7.3 (5.0,10.3)
How many times have you traveled outside your village in the past 6 months?			
0-5 times	2.0 (1.4,2.7)	6.9 (5.9,8.1)	8.6 (7.5,9.9)
6+ times	0.6 (0.1,1.8)	2.5 (1.3,4.4)	3.2 (1.8,5.2)
Frequency of chores that involve trips to water			
Often (almost every day)	2.4 (1.7,3.1)	7.2 (6.0,8.4)	9.2 (7.9,10.6)
Rarely (special cases, e.g. burial)	0.4 (0.1,1.0)	4.1 (2.8,5.7)	4.3 (3.0,5.9)
Frequency of chores that involve trips to forest			

Often (almost every day)	3.0 (2.1,4.2)	8.2 (6.7,9.9)	10.7 (8.9,12.6)
Rarely (special cases, e.g. burial)	0.8 (0.4,1.4)	4.8 (3.8,6.0)	5.5 (4.5,6.8)
Do you use this mosquito net regularly (at least 4-5 nights per week)?			
No	2.5 (1.2,4.6)	7.6 (5.2,10.7)	9.1 (6.5,12.4)
Yes	1.6 (1.1,2.2)	6.0 (5.0,7.0)	7.4 (6.4,8.6)
Residence (Have you lived in this village for > 6 months?)			
No	0.0 (0.0,1.9)	4.1 (1.8,8.0)	4.1 (1.8,8.0)
Yes	1.9 (1.4,2.5)	6.4 (5.4,7.4)	8.0 (6.9,9.1)
Headache			
No	1.3 (0.8,2.1)	5.5 (4.3,6.9)	6.8 (5.5,8.2)
Yes	2.1 (1.4,3.1)	7.0 (5.7,8.4)	8.7 (7.2,10.3)
Body ache/pain			
No	1.3 (0.8,2.0)	5.8 (4.6,7.1)	6.8 (5.6,8.2)
Yes	2.4 (1.6,3.4)	6.8 (5.5,8.4)	8.9 (7.3,10.6)
Nausea			
No	1.7 (1.2,2.2)	6.1 (5.2,7.1)	7.5 (6.5,8.6)
Yes	2.5 (1.0,5.1)	7.5 (4.7,11.2)	9.6 (6.5,13.7)
Vomiting			
No	1.6 (1.2,2.2)	6.1 (5.1,7.1)	7.4 (6.4,8.6)
Yes	2.7 (1.1,5.4)	7.6 (4.7,11.5)	9.9 (6.6,14.2)
Abdominal discomfort			
No	1.8 (1.3,2.5)	6.1 (5.1,7.3)	7.7 (6.5,8.9)
Yes	1.5 (0.8,2.7)	6.5 (4.9,8.5)	7.8 (6.0,9.9)
Decreased appetite			
No	2.0 (1.4,2.7)	5.9 (5.0,7.0)	7.6 (6.5,8.8)
Yes	1.0 (0.4,2.1)	7.2 (5.3,9.5)	8.1 (6.1,10.5)
Fatigue			
No	1.8 (1.3,2.5)	5.9 (5.0,7.0)	7.4 (6.3,8.6)
Yes	1.5 (0.6,2.9)	7.4 (5.3,10.0)	8.9 (6.6,11.6)
Fever with chill and rigor			
No	1.6 (1.2,2.2)	6.1 (5.2,7.1)	7.5 (6.5,8.7)
Yes	2.5 (1.1,4.8)	7.1 (4.5,10.4)	8.9 (6.1,12.6)
In the past 24 hours, have you had a fever?			
No	1.8 (1.3,2.3)	6.2 (5.3,7.2)	7.7 (6.7,8.8)
Yes	1.0 (0.0,5.7)	7.3 (3.0,14.4)	8.3 (3.7,15.8)
Febrile (Temperature 99.5F)			
No	1.7 (1.2,2.3)	6.1 (5.2,7.1)	7.6 (6.6,8.7)
Yes	3.0 (0.4,10.4)	10.4 (4.3,20.3)	11.9 (5.3,22.2)
State/Region			
Laiza Site	0.0 (0.0,0.4)	9.6 (7.9,11.6)	9.6 (7.9,11.6)
Ann Site	4.5 (3.3,6.0)	5.8 (4.4,7.4)	9.6 (7.8,11.6)
Monywa Site	0.3 (0.0,1.0)	2.0 (1.1,3.3)	2.3 (1.3,3.6)
Total	1.7 (1.3,2.3)	6.2 (5.3,7.2)	7.7 (6.7,8.8)

Supplemental Table 5. Sensitivity analysis results of regression models assessing the correlates of baseline sub-clinical malaria infection and the rate of repeated sub-clinical infection status over time, complete case and multiple imputed – excluding Monywa Site

	Baseline Prevalence N=1,996		Count of Positives (Complete Case) N=140		Count of Positives (Multiple Imputation) N=192	
**Independent Variable**	**Baseline Risk (RR) All Malaria**	**P-value**	**Complete Case IRR (95% CI)**	**P-value**	**Multiple Imputation IRR (95% CI)**	**P-value**
Age, n(%)		.		.		.
0.5-16	(ref)	.	(ref)	.	(ref)	.
17-40	1.29 (0.93,1.80)	0.129	0.59 (0.37,0.94)	0.026	0.78 (0.64,0.96)	0.021
41+	1.62 (1.15,2.30)	0.006	0.58 (0.36,0.92)	0.022	0.75 (0.61,0.93)	0.009
Sex						
Male	(ref)	.	(ref)	.	(ref)	.
Female	0.63 (0.48,0.83)	0.001	1.01 (0.72,1.41)	0.966	1.02 (0.91,1.15)	0.722
Type of main occupation		.		.		.
Indoor	(ref)	.	(ref)	.	(ref)	.
Outdoor	2.66 (1.88,3.76)	0.000	0.73 (0.46,1.18)	0.197	0.67 (0.55,0.82)	0.000
What is your main occupation?		.		.		.
Dependent	(ref)	.	(ref)	.	(ref)	.
Student	2.21 (1.30,3.76)	0.004	1.27 (0.58,2.80)	0.549	1.41 (1.05,1.90)	0.024
Soldier	3.54 (1.40,8.93)	0.007	0.96 (0.28,3.32)	0.945	0.90 (0.58,1.37)	0.607
Refugee	3.80 (1.91,7.55)	0.000	0.74 (0.32,1.69)	0.471	0.85 (0.63,1.15)	0.287
Farmer	2.89 (1.52,5.49)	0.001	0.96 (0.39,2.39)	0.931	1.00 (0.73,1.39)	0.979
Plantation worker	4.09 (2.23,7.51)	0.000	0.89 (0.36,2.21)	0.798	0.99 (0.72,1.35)	0.938
Other	3.46 (1.79,6.69)	0.000	1.01 (0.39,2.64)	0.977	1.12 (0.80,1.56)	0.520
How far away is your place of work or study?		.		.		.
<1 km	(ref)	.	(ref)	.	(ref)	.
1-5 km	1.93 (1.43,2.62)	0.000	1.05 (0.71,1.57)	0.792	1.02 (0.86,1.20)	0.849
>5 km	2.30 (1.44,3.65)	0.000	1.16 (0.60,2.26)	0.658	1.07 (0.83,1.39)	0.577
What mode of travel do you use most frequently when you travel to work or school?		.		.		.
Non-motorized	(ref)	.	(ref)	.	(ref)	.
Motorized	1.14 (0.75,1.73)	0.534	0.63 (0.39,1.00)	0.051	0.71 (0.55,0.90)	0.006
Work at home	0.40 (0.26,0.60)	0.000	1.13 (0.63,2.03)	0.692	1.03 (0.83,1.27)	0.804
Do you stay overnight for work or study?		.		.		.
No	(ref)	.	(ref)	.	(ref)	.

Yes	1.20 (0.84,1.72)	0.318	0.97 (0.65,1.44)	0.861	1.08 (0.91,1.28)	0.393
Did your main occupation vary seasonally in the past one year?		.		.		.
No	(ref)	.	(ref)	.	(ref)	.
Yes	1.45 (0.97,2.17)	0.073	0.84 (0.51,1.37)	0.482	0.83 (0.66,1.04)	0.098
How far is the greatest distance that you travel in a typical day?		.		.		.
<1 km	(ref)	.	(ref)	.	(ref)	.
1-5 km	1.88 (1.38,2.57)	0.000	1.12 (0.75,1.65)	0.584	0.98 (0.84,1.14)	0.774
>5 km	2.61 (1.63,4.19)	0.000	1.03 (0.60,1.76)	0.915	0.87 (0.69,1.10)	0.254
How many times have you traveled outside your village in the past 6 months?		.		.		.
0-5 times	(ref)	.	(ref)	.	(ref)	.
6+ times	0.70 (0.32,1.54)	0.375	0.91 (0.43,1.94)	0.811	0.90 (0.66,1.22)	0.488
Frequency of chores that involve trips to water		.		.		.
Often (almost every day)	(ref)	.	(ref)	.	(ref)	.
Rarely (special cases, e.g. burial)	0.49 (0.33,0.71)	0.000	0.88 (0.58,1.32)	0.533	0.95 (0.81,1.11)	0.518
Frequency of chores that involve trips to forest		.		.		.
Often (almost every day)	(ref)	.	(ref)	.	(ref)	.
Rarely (special cases, e.g. burial)	0.57 (0.42,0.76)	0.000	1.28 (0.89,1.86)	0.181	1.21 (1.03,1.42)	0.020
Do you use this mosquito net regularly (at least 4-5 nights per week)?		.		.		.
No	(ref)	.	(ref)	.	(ref)	.
Yes	0.76 (0.53,1.09)	0.137	1.27 (0.79,2.02)	0.323	1.21 (1.01,1.44)	0.039
Residence (Have you lived in this village for > 6 months?)		.		.		.
No	(ref)	.	(ref)	.	(ref)	.
Yes	1.53 (0.69,3.41)	0.293	1.16 (0.56,2.37)	0.692	1.24 (0.87,1.78)	0.227
Timing of baseline data collection		.		.		.
Peak (Jun-Dec)	(ref)	.	(ref)	.	(ref)	.
Non-Peak (Jan-May)	1.06 (0.81,1.40)	0.656	0.88 (0.60,1.30)	0.525	0.96 (0.83,1.11)	0.552
usPCR result						
P. vivax positive at baseline		.	0.65 (0.39,1.09)	0.102	0.72 (0.59,0.89)	0.002
P. falciparum positive at baseline		.	1.75 (1.04,2.93)	0.035	1.38 (1.11,1.73)	0.005
Self-reported symptoms (past 2 months), n(%)						
Headache	1.15 (0.86,1.54)	0.343	1.25 (0.90,1.75)	0.184	1.14 (0.98,1.34)	0.094
Body ache/pain	1.33 (0.97,1.82)	0.074	1.28 (0.91,1.80)	0.162	1.31 (1.14,1.50)	0.000
Nausea	1.22 (0.81,1.83)	0.335	1.39 (0.76,2.54)	0.284	1.31 (0.98,1.73)	0.064
Vomiting	1.29 (0.86,1.94)	0.222	1.62 (0.82,3.17)	0.163	1.60 (1.21,2.11)	0.001
Abdominal discomfort	1.00 (0.74,1.33)	0.976	1.09 (0.74,1.60)	0.662	1.16 (1.00,1.34)	0.051
Decreased appetite	0.94 (0.69,1.29)	0.713	1.01 (0.67,1.52)	0.952	1.10 (0.91,1.31)	0.318

Fatigue	1.12 (0.81,1.55)	0.481	0.83 (0.57,1.20)	0.316	0.96 (0.82,1.12)	0.588
Fever with chill and rigor	1.24 (0.83,1.84)	0.294	1.31 (0.71,2.43)	0.389	1.23 (0.96,1.58)	0.102
Self-reported symptoms (past 24 hours)						
In the past 24 hours, have you had a fever?	1.06 (0.52,2.16)	0.871	0.88 (0.37,2.10)	0.771	1.01 (0.66,1.53)	0.975
Measured clinical characteristics						
Temperature (F)(Standardized)	1.16 (1.02,1.33)	0.029	1.09 (0.90,1.33)	0.371	1.04 (0.95,1.14)	0.381
Febrile (Temperature 99.5◦F)	1.53 (0.79,2.98)	0.211	1.13 (0.49,2.65)	0.770	1.21 (0.82,1.79)	0.330
Hemoglobin (standardized)	1.11 (0.94,1.31)	0.238	0.99 (0.82,1.19)	0.895	1.00 (0.94,1.07)	0.990
